# Developing a community-based nursing and midwifery career pathway – A narrative systematic review

**DOI:** 10.1371/journal.pone.0211160

**Published:** 2019-03-28

**Authors:** Clare Harvey, Desley Hegney, Agnieszka Sobolewska, Diane Chamberlain, Elspeth Wood, Lisa Wirihana, Sandy Mclellan, Joyce Hendricks, Troy Wake

**Affiliations:** 1 School of Nursing, Midwifery and Social Science, Central Queensland University, Townsville campus, Townsville, Queensland, Australia; 2 Research Division, Central Queensland University, Brisbane Campus, Brisbane, Queensland, Australia; 3 The Nursing School, University of Adelaide, Adelaide, South Australia, Australia; 4 College of Nursing & Health Sciences, Flinders University, Adelaide, South Australia, Australia; 5 School of Nursing, Midwifery and Social Science, Central Queensland University, Noosa Campus, Noosa, Queensland, Australia; 6 School of Nursing, Midwifery and Social Science, Central Queensland University, Mackay campus, Mackay, Queensland, Australia; 7 School of Nursing, Midwifery and Social Science, Central Queensland University, Bundaberg Campus, Bundaberg, Queensland, Australia; 8 Mackay Health Service, Department of Health, Proserpine, Queensland, Australia; Aga Khan University, KENYA

## Abstract

**Introduction:**

Community nursing and midwifery is changing in response to a shift in care from hospital to home, brought about by increasing costs to care because of an aging population and increasing chronicity. Until now, community nursing positions and scope of practice has been dependent on service focus and location, which has led to the role being unclearly defined. Lack of appeal for a career in community practice and a looming workforce shortage necessitates a review into how community nursing and midwifery transition to practice is supported.

**Methods:**

This review sought to identify, assess and summarize available evidence relating to transitioning into community nursing and midwifery practice as a speciality. A systematic review was conducted using the Preferred Reporting Items for Systematic Reviews and Meta-Analyses approach. A narrative synthesis was then undertaken on papers that examined community nursing and midwifery pathway perspectives which define, and enable or inhibit a contemporary pathway. Thematic analysis used a theoretical framework developed for early career and rapid transition to nursing specialty practice.

**Results:**

There is a paucity of research that identifies community nursing and midwifery as a discreet scope of practice. Twelve papers were eventually included in the review. Verbatim findings were extracted from the papers and clustered into categories based on the chosen theoretical framework. Major themes were ‘the self’ (professional and personal); ‘transition processes’; and, a ‘sense of belonging’. Sub themes included narrative identifying inhibitors and enablers in each theme.

**Discussion:**

No definition of community practice or pathway was identified in nursing, although midwifery was clearly defined. Community nursing practice was described as generalist in nature although specialist knowledge is required. Being part of the community in the professional sense and personal sense was considered important. The importance of transition was identified where pre-entry exposure to community practice was seen as important. Stages in transition to practice were recognised as pre-entry; incomer; insider; and, a sense of belonging. The process of transition should be planned and individualised acknowledging past experience whilst acknowledging the specialist nature of community-based practice.

## Introduction

The terms primary health care and community care are often used interchangeably, with the concepts encompassing care that is “accessible, acceptable, affordable and equitable and delivered close to where people live, work and play” [1]. The focus of primary health care is principally on early detection of illness, health promotion and early intervention, in line with the Alma Ata Declaration [2]. Primary care is also the first level of contact for people entering the health care system [3]. In Australia, all three concepts are used as care that is provided in the community, through primary health networks, community-based hospital services and non-government organisations, all aim at improving the health of the population [4].

Community nurses and midwives are flexible, autonomous, able to adapt care to the service delivery setting, and have a diversity of knowledge and skills [5]. The definitions of community nursing are not static and are influenced by how the role has changed over time. This is in response to important healthcare fluctuations and reform that have tested the scope of the community nurse and midwife. These include: (1) Changes to the acute care sector, obstetric and midwifery services that have resulted in the limited availability of hospital beds, early client discharge and increased in home care [6, 7]. (2) An aging population with more complex often chronic health issues, coupled with the complex social conditions of today [8]. (3) Greater implementation of illness prevention and health promotion programs [9]. (4) The focus on preventing deterioration often in chronic health conditions in order to reduce hospitalisation [10]. Although community nurses and midwives have adapted over time, this has led to a diminution of the specificity of the role and therefore a current accurate definition. The World Health Organisation (WHO) defined community nursing in 1974 [11] as;

*“a special field of nursing that combines the skills of nursing, public health and some phases of social assistance and functions as part of the total public health programme for the promotion of health, the improvement of the conditions in the social and physical environment, rehabilitation of illness and disability”*,

and still uses this definition in current reports [12], despite the vast changes to the role and scope of practice that contemporary community practice demands. In Australia, the Australian Primary Health Care Nurses Association (APNA) compartmentalises community nursing into three categories: (1) care-in-home for those who are disadvantaged with the nurses being conduits between hospital and home; (2) correctional service nurses who work with incarcerated patients; and (3) practice nurses who work in primary health networks under the direction of general practitioners [13]. While these definitions may capture some of the context of practice, geographical location of practice is not addressed by APNA, nor is the assumption of the specialisation of community nursing which the WHO definition still endorses. Midwives on the other hand, are considered a specialty, by the very nature of their practice, yet community midwives are not prevalent in Australia [14].

Midwifery has taken a different professional career path to nursing over the last 20 years, with midwives in Australia being separately registered and educationally prepared [15, 16]. Midwives are not commonly located in community-based practice with the vast majority of midwives working in hospitals [15]. Although primary maternity units have demonstrated cost effectiveness, the smaller rural and remote communities do not have the birthing numbers to sustain such practices [17]. Yet, it is acknowledged that women receiving care from midwifery group practices have enhanced antenatal and postnatal care with significant costs savings identified [18].

Community nursing and midwifery has also become less visible than other health care services or specialities due to: (1) the geographical location from metropolitan, to regional and rural and remote communities [5]; (2) the shared responsibilities with other health professionals such as allied health and general practitioners [19, 20]; (3) complexity of social and health issues that require the development of referral pathways [21]; (4) the funding differences for community practice compared to acute care services [22, 23]. In Australia, community nursing and midwifery manage care across different funding boundaries, the latter often leaving community services with less capital to manage complex care. Funding for current care delivery models do not support integrated care when several specialist services are visiting the patient, a matter that questions the cost savings of the home care of co- and multi-morbid clinical presentations [24].

Primary care services are predominately provided by general practitioners who receive remuneration through the Federal government’s “Medicare” universal health care system [25]. Other health services in rural areas are influenced by the size and distance from a major centre and range from regional hospitals to single nursing posts. In most cases these nurses are employed by the State government and work with on or off-site multidisciplinary teams. In the twenty-first century few nurses work solely in the community, rather they are employed and work from a health service [26].

Many countries including Australia are facing a nursing and midwifery workforce crisis, with more than half of the workforce now over 45 years of age [27]. In 2015, the Queensland Government committed funding to increase the employment of new graduates to support and transition into the general nursing and midwifery workforce, and into speciality areas of practice. Community nursing and midwifery are two of those identified specialities. Currently, in Australia, there is a lack of standardisation for community nursing and midwifery [28]. Not only in the definition of the specialties, but also in the defined scope of practice [29]. Also, the educational and experiential preparation for community nursing and midwifery specialisation is not overt and needs development [30, 31].

Therefore, the aim of this review is to: (1) define community nursing and midwifery within the context of contemporary specialist practice in Australia. (2) Formulate for the Queensland government both an early career transition pathway for community nursing and midwifery as the first phase of a three-phase study.

The research questions for this review are:

1. What defines a community nurses/midwife scope of practice, its jurisdiction of practice and its specialty areas in practice?
2. What career pathways currently exist for community nursing and midwifery practice and do these require modification?
3. What education and clinical preparation is required for community nursing and midwifery development?
4. What are the preferred models of community nursing and midwifery preparation for practice, and how can these be implemented to encourage nurses’ and midwives’ entry into community practice, and their retention in the community nursing and midwifery workforce?

## Method

### Approach

We adopted the Preferred Reporting Items for Systematic Reviews and Meta-Analyses approach [32] to systematic review and the work of Popay and colleagues [33, 34] in conducting this systematic review. The protocol of this systematic review is registered with the PROSPERO [35] International Prospective Register of Systematic Reviews: CRD42018100228 [36]. We conducted a narrative synthesis of available papers that examined community nursing and midwifery pathway perspectives of the factors which define, and enable or inhibit a contemporary career pathway. Narrative synthesis is regarded as an effective way to identify the story underpinning a disparate body of evidence by giving reviewers the flexibility to develop themes that bring coherence to that data. This approach was considered particularly useful to examine themes related to factors perceived to inhibit or enable community nursing or midwifery career pathways.

The conceptual framework thematic analysis will be based on the theoretical framework of the “Effective early career and rapid transition to a nursing specialty in differing contexts of practice (TRANSPEC)” [37]. Hegney et al. [37], define specialist practice as:

*“a practice that follows and builds on generalist practice. It focuses on a specific area of nursing and is directed towards a defined population or a defined area of activity and it is reactive of depth of knowledge and relevant skills*.*” (p. 4)*

Specialist practice may occur at any point in time on a continuum from beginning to advanced, and is referred to as the transition period. Hegney et al. [37], TRANSPEC theoretical framework includes the following themes of the specialist transitioning clinician appropriate to community practice. The Self–professional and personal; the Transitioning Processes–formal and informal; a Sense of Belonging to the team, organisation and/or community; and the Context of Practice. Enablers and inhibitors within these four areas influence transition at three major points: pre-entry; immediately on entry (incomer) and when established in the speciality (insider). The conceptual framework thematic analysis of this review will be constructed from this transitioning theoretical model [37].

### Selection criteria

**Fig 1:**
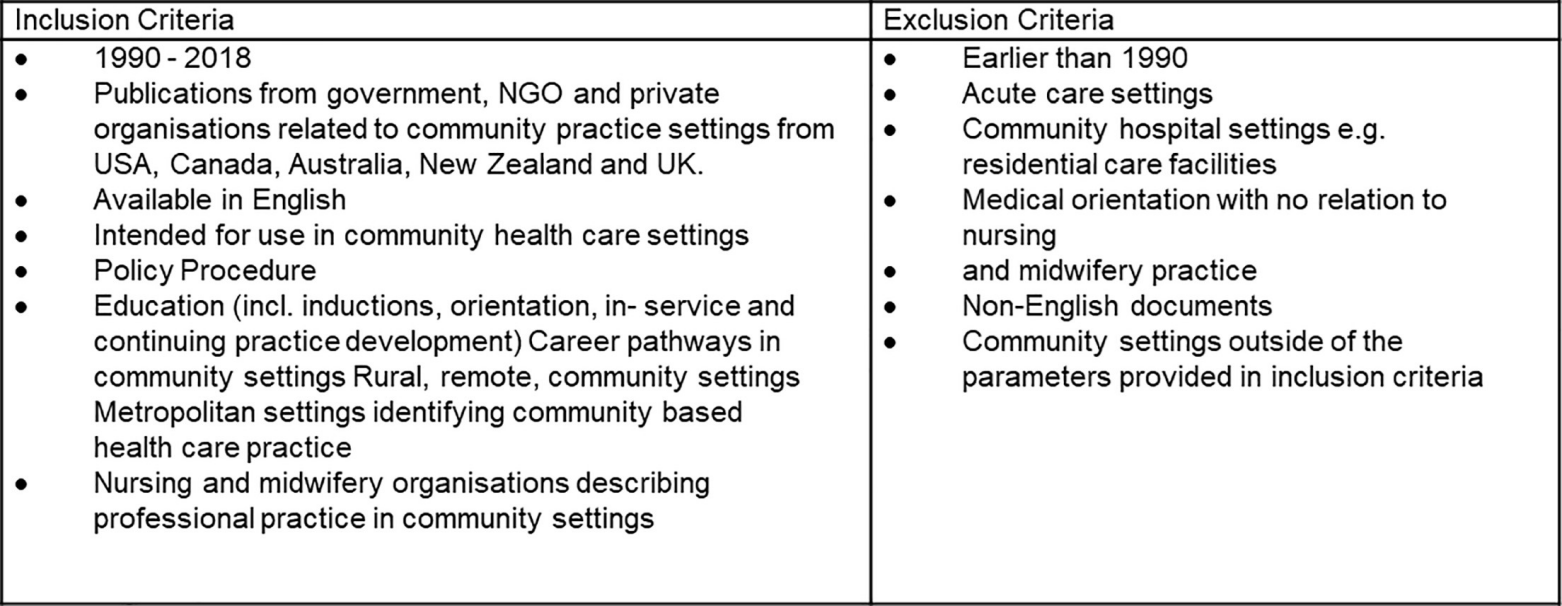
Search inclusion and exclusion criteria.

The inclusion criteria included primary research studies published in peer reviewed English language journals published in Australia, New Zealand, United Kingdom, Canada and the United States of America. Our population for this search were nurses and midwives who worked in community-based settings. We included metropolitan, rural and remote contexts of practice with a focus on transition to practice related to education, orientation, induction, mentorship and coaching activities. Inclusion and exclusion criteria are provided in Fig 1.

### Search strategy

A systematic search was conducted in June 2018. Eight electronic databases (CINAHL Plus, MEDLINE, Scopus, Web of Science and ProQuest and PsycInfo, Embase, JBI and Cochrane electronic databases) were searched from January 2000 to July 2018, with no language restrictions. We attempted a search from January 2008 to July 2018 but there was a distinct lack of relevant literature and we needed to extend the search back further to 2000. Any older literature would not represent current evidence. The following subject heading and keywords were used: (Population/Context “community health nurs*”, OR “primary care nurs*” OR “district nurs*” OR “public health” OR “community health care” OR “domiciliary health care” OR “domiciliary care” OR “community nurs*” AND “midwifery” AND “community health nurs*” AND “recruit” OR “retain” OR “selection” OR “career*” OR “appoint*” OR “education*” OR “specialty*” OR “retain*” OR “attrition”). In addition, reference lists of included studies and reviews were checked for further possible studies.

The results from the searches were imported into EndNote X8 to remove duplicates and to manage the references through the different stages of the review. Titles and abstracts of studies retrieved using the search strategy were screened independently by four authors to identify studies that potentially met the inclusion criteria. Data were extracted and documented using an extraction form developed to identify relevant information.

### Critical appraisal

**Fig 2:**
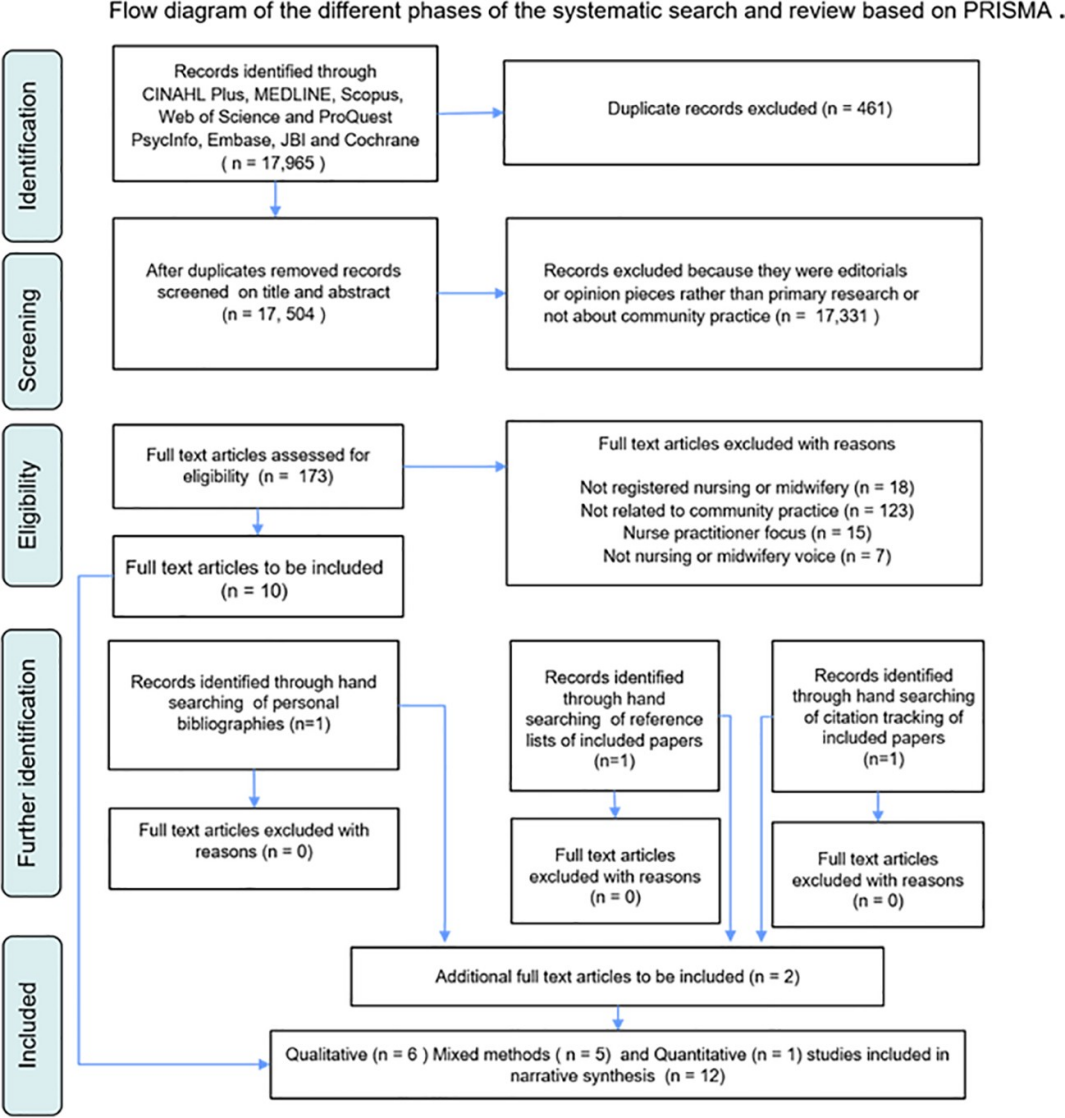
Flow diagram of the different phases of the systematic search and review based on PRISMA [23].

The quality of the relevant literature was appraised by two authors who independently assessed for risk of bias. Due to the lack of randomized controlled trials, and the predominance of surveys, quantitative papers were critically appraised using the Critical Appraisal Questions for Surveys (CAQS) tool [38] with a cut-off score of six out of 10. Reasons for exclusion were: insufficient information on recruitment processes; measurement bias unclear; confounding factors in design and/or analysis unclear; results flawed; insufficient discussion of implications for transferability or generalizability. For assessing the quality of qualitative studies, the Joanna Briggs Institute's (JBI) Qualitative Assessment and Review Instrument (QARI) [39] with ten criteria was used. Quality was quantified by calculating scores of either 0 or 1 point per criterion. Reasons for exclusion were: lack of congruity between the research methodology and the methods used to collect the data; lack of congruity between the research methodology and the interpretation of results; the influence of the researcher on the research; participants and their voices being adequately represented; conclusions drawn in the research report that flowed from the analysis, or interpretation, of the data. To be selected for this review, original studies had to fulfil at least six assessment criteria. Based on the quality appraisal, 12 studies were included in this review. Refer to Fig 2 for the PRISMA reporting items of the search.

## Results

**Fig 3:**
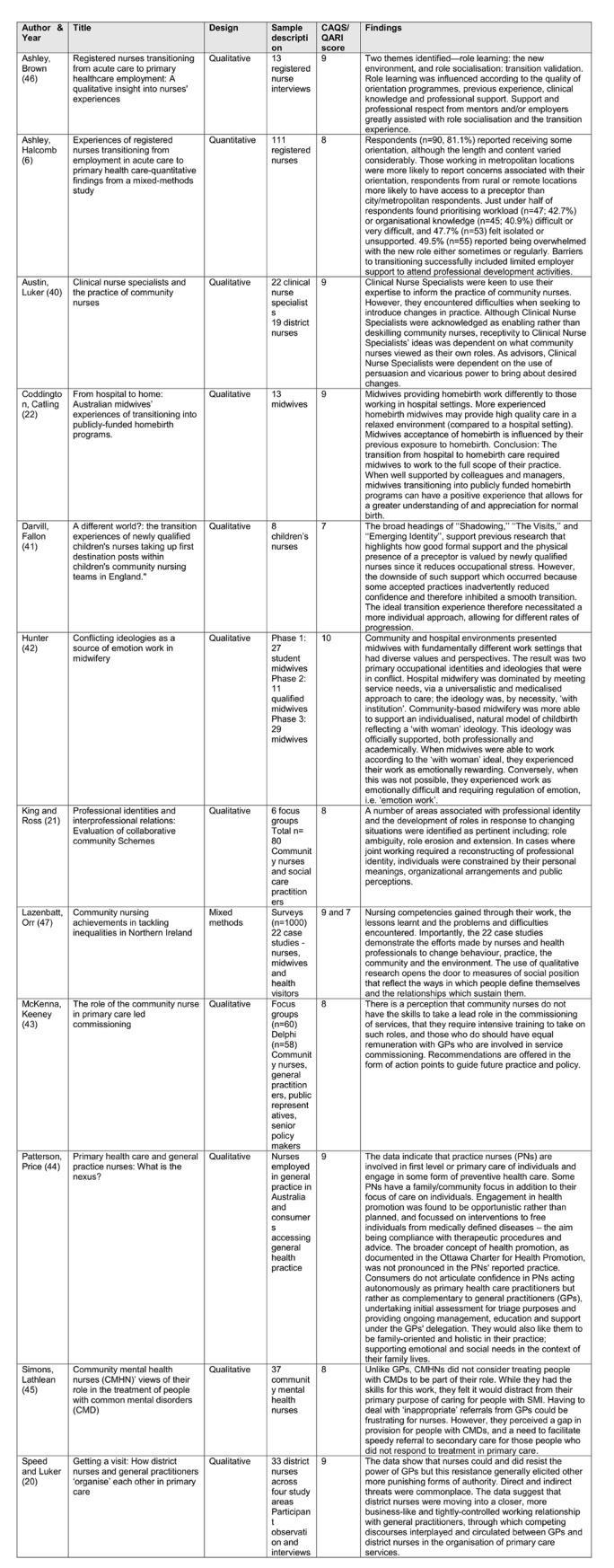
Articles included in this review.

There has been relatively little research conducted specifically in relation to community nursing and midwifery when compared to other areas of nursing/midwifery. Searches of databases and bibliographies yielded 17,965 potentially relevant citations, of which 461 were duplicates and 17, 331 were deemed ineligible on the basis of title and abstract. Full text was retrieved for 173 studies and these papers were assessed against the inclusion and exclusion criteria. A final sample of 12 studies was included in the review. There were ten qualitative papers [20–22, 40–46] and one mixed methods [47] and one quantitative paper [6]. The types of papers and the roles of the participants in the studies are outlined in Fig 3. Also provided in this figure is the verbatim findings extracted from the papers to identify the importance of the narrative, regardless of research design used for data collection and analysis [34].

### Narrative synthesis and conceptual framework

Narrative synthesis was arranged into a thematic structure and conceptual framework based on the TRANSPEC theoretical framework of Hegney et al. [37], for the “Effective early career and rapid transition to a nursing specialty in differing contexts of practice”. Narrative descriptions were extracted verbatim from articles and clustered into categories that formed three major themes described as ‘the self’ (personal and professional); ‘transition processes’; and ‘sense of belonging’. Sub-themes created within the narrative were divided into inhibitors and enablers. Themes were coded and re-coded by four members of the research team, with the final selection being approved by consensus from all research members (Fig 3). There were also three different time points in the transition process that were identified: pre-entry, incomer and insider to the transition.

During the analysis, sub-themes emerged crossing both boundaries of inhibitors and enablers with either the negative or positive reflections being provided (Fig 3). With the exception of Ashley et al. and Darvill et al., [6, 41], there was no reference to new graduate or pre-entry into community practice. Reference to new community nurses predominantly focused on nurses transitioning from acute care [46].

### Definition of the scope of community practice

A definition of community practice was not evident in the nursing practice areas; however, midwifery was clearly defined, being one single practice of care for mothers and babies [42, 48]. Although being part of the community was identified, there were variances of definition, with a clear distinction highlighted between being part of the community in a professional sense, and being part of the community in the sense of living within a particular community, in the personal sense. The idea of ‘generalist’ was evident [44, 47, 49, 50, 51], yet nurses verbalised the need for specialist knowledge [40, 45, 51].

### What career pathways currently exist for community nursing and midwifery practice and do these require modification?

We found no clear career pathways in the published literature. We have constructed a conceptual Community Nursing and Midwifery Career Pathway Framework using the narrative thematic analysis aligning with the themes of the TRANSPEC theoretical framework.

S1 Fig presents the thematic analysis findings of the review. Using the TRANSPEC theoretical framework of Hegney et al., the self, transition processes and a sense of belonging were the major themes within the context of practice. Each of the enablers and inhibitors impacted on these themes at three phases: pre-entry, incomer and insider.

**Fig 4:**
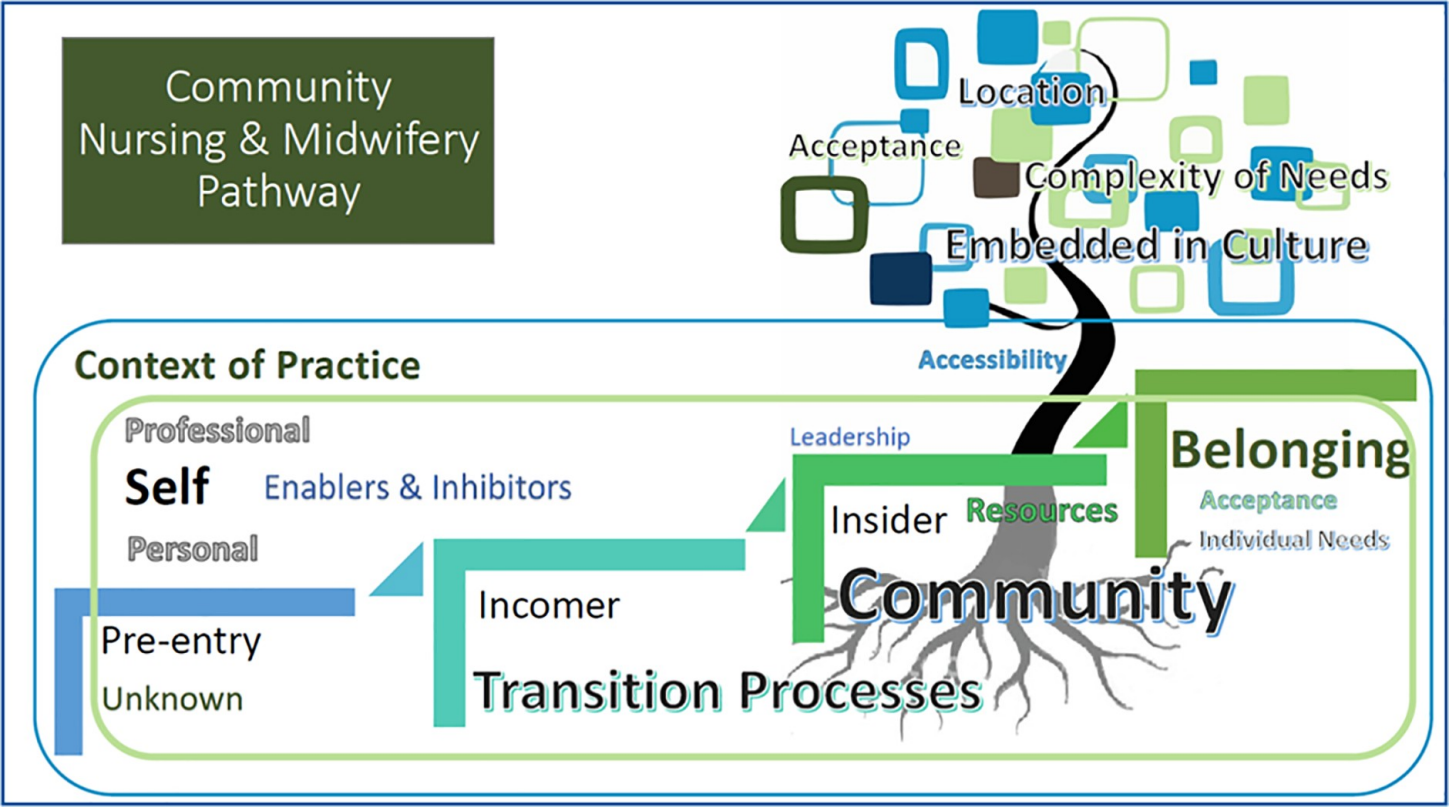
The community nursing and midwifery career pathway framework.

Fig 4 represents the thematic findings from the review schematically designed into the Community Nursing and Midwifery Career Pathway.

The self as a theme is comprised of the personal and the professional self and continues throughout the pathway. The transition processes refer to the formal and informal processes that support transition. A Sense of Belonging relates to the nursing and midwifery practice around the sub-themes of acceptance, location, complexity of needs, embedded in culture and accessibility. The emerging themes and sub-themes occur in the presence of enablers and inhibitors. The enablers and inhibitors can be related to the self, the transition process and the community. The emerging concepts are interrelated.

### The community nursing and midwifery career pathway framework

The Community Nursing and Midwifery Career Pathway framework of a transitioning nurse or midwife in a Community Practice includes PRE-ENTRY (exposure to community-based practice though structured clinical placements and education prior to commencing in community nursing or midwifery practice), through to INCOMER (beginning work and therefore beginning a transition to practice either as a new graduate or experienced professional). Then to an INSIDER (as a professional embedded and accepted in and by the community and working at novice specialist within a community-based setting). The main premise of the pathway is that transition is an individual process which takes place in the context of both the immediate community workplace or health service and the local community. The tree starting with the roots, represents the community and the journey of practice to embeddedness in a community setting. Resources, leadership, accessibility, culture, location, acceptance leading to a sense of belonging of the individual clinician are sub-themes that impact on the incomer’s preparation for practice.

#### What education and clinical preparation is required for community nursing and midwifery development?

The education and clinical preparation evidence was minimal within this systematic review and aligns with the theme of pre-entry. Areas of pre-entry deficiency are outlined in the model and requires further investigation.

#### What are the preferred models of community nursing and midwifery preparation for practice, and how can these be implemented to encourage nurses’ and midwives’ entry into community practice, and their retention in the community nursing and midwifery workforce?

There were no specific models of practice identified in the review. Preparation to practice in terms of orientation and learning opportunities were ad hoc and focused on service related activity rather than actual transition to practice. The need to provide a pathway and framework for community-based practice orientation, transition and ongoing learning is needed.

## Discussion

The aims of this systematic review were to identify, assess and summarize available evidence relating to transitioning into a community nursing practice as a speciality. This systematic review provides a new conceptual framework and model for community career pathway for specialist nurses. A conceptual framework was developed from the findings of this systematic review.

The main themes identified in the transition process included: the self (personal and professional); the transition processes (formal, informal); and a sense of belonging to the team and the organisation. It was seen that there were four stages to the transition which were: pre-entry, incomer, insider, and a sense of belonging. Determinants of successful transition at each stage were identified in terms of enablers and inhibitors.

As Fig 4 illustrates, transition has been described as the period of time when a new staff member adapts and integrates into a new clinical setting, inclusive of organisational requirements, and in the development of pertinent clinical skills [52, 53]. Wagner [54] argues that learning is a social practice that is achieved through active engagement with the environment within where the learning takes place as a combination of social assimilation and individual experience. Nurses entering a new practice require the support from organisation in relation to orientation and the allocation of mentors [55, 56]. The main premise of the pathway is that transition is an individual process which takes place in the context of individual workplace and the local community.

This systematic review confirmed lack of census around standardisation and scope of practice of nurses and midwives in community practice [28, 29]. In comparison to nurses, midwives in the community have clear professional boundaries [42]. There were variance of definition in community nursing practice. The idea of ‘generalist’ was evident [44, 47], yet nurses in the community verbalised the need for specialist knowledge [40, 45]. Lack of role definition of community practice lead to role ambiguity and confusion [5, 19, 43, 45, 57–59].

Similar to a systematic review conducted by Hegney et al. [37], this systematic review also highlighted that the current knowledge was deficient in the pre-entry phase. This is a phase which determines the attraction and recruitment to the community speciality. Although early and rapid career transitioning to community nursing has been suggested as a strategy [52], nurses and midwives in community practice are more likely to be expected to work as a sole practitioner with more autonomy than their hospital based colleagues [41]. New graduates are less likely to be equipped with such skills. This is when transitioning individuals feel like they are *“thrown into [the] deep end”* [46]. An innovative strategy has commenced in Australia for community nursing students. Hartman [60] implemented a simulation laboratory in home health care settings which were well received by students and assist the preparation of transitioning students into their community practice.

Transitioning processes should be individualised as suggested by Darville et al. [41], where a standardised framework and guidelines needs to be available for formal transitioning processes. Such a guideline should be structured in a way to allow for transfer to various workplace settings and flexible enough to meet the needs of individuals. For example, length of supervision should depend on the confidence and competence of transitioning individuals [22, 41, 46].

The evidence also highlighted that transitioning individuals desired the need to learn new skills beyond their nursing or midwifery practice. Transitioning individuals need to be able adapt to new community practice settings, different to the hospital context [41, 46],from being a member of healthcare team to a sole practitioner [46]. In particular, this is evident for nurses who are working in general practices [46]. Some transitioning individuals acknowledge such support in the workplace [22, 46] whilst others face challenges in adapting to being a sole practitioner [45, 46]. A strategy such as the development of exchange programs between a community nurse/midwife and hospital-based nurse/midwife might assist with this adaption. It would enhance the collaboration between hospital and community healthcare services, similar to well established transitioning programs to nursing specialities [61, 62]. Such opportunities would allow community nurses and midwives to upskill and adapt their clinical skills whilst allowing others to explore community nursing/midwifery as a potential career change. This would also provide an experience to assist decision making of new graduates who want to enter community nursing/midwifery. Programs such as hospital outreach model is another option which allows nurses and midwives to be affiliated with a hospital and provide their care in the community setting. Such a model of practice is evident in midwifery where participants of studies included hospital based and self-employed individuals [48, 63].

This review focused on perspectives of nurses and midwives transitioning to the community setting. Other studies have examined this issue from differing perspectives. Patterson et al. focused on the consumers’ viewpoints about their needs of community nursing [44]. Other studies have surveyed nurses transitioning to rural practice, which if different in context, also concludes that an appropriate orientation and supervision support enhances a positive transitioning experience [50, 56, 64–66]. In addition, Lea and Cruickshank have also highlighted the importance of organisational support and leadership as important for transitioning new graduates to rural and remote practices [56, 65]. These results suggest that transitioning to a rural and remote practice might share similar transitioning experience to the community practice.

### Limitations

We found a significant deficiency in the literature specific to our questions. There was a paucity of evidence about the whole process of transitioning to community practice. Our methods were constructed around the limited literature that was available. Therefore, in response to this we developed a conceptual model from the TRANSPEC theoretical framework to lead the development of a new Community Nursing and Midwifery Career Pathway for further research.

## Recommendations

This review could not determine a contemporary definition of community nursing, with only a limited definition of midwifery community practice evident. The whole spectrum of community practice needs to be examined carefully to structure a definition before recruitment or retention can be successful. These are where the deficiencies are evident on our framework and model.

The review has highlighted the lack of evidence at the pre-entry phase. Further research should focus on the impact of targeted marketing programs in the pre-entry time point. Likewise, the access to community practice clinical placements during pre-entry and the impact of a formal transitioning program and exchange programs to community practice needs investigation.

The assumption that community practice and skills is an extension of acute care practice is evident but not supported in this review. Further work is needed to establish what educational preparation, skill and scope of practice are required in the community setting.

## Conclusions

We have conducted a systematic review that provides a new conceptual framework and model for the transition of nurses and midwives to a specialist community practice career. We used the TRANSPEC model as a theoretical framework for specialist practice due to a lack of a contemporary definition of community nursing and midwifery as specialist practice.

The transition model sets out the multiple and complex factors that come into play only as this review provides a preliminary understanding of a successful career pathway for nurses and midwives into community practice.

We believe further research into the factors influencing pre-entry and achieving a sense of belonging into community practice is essential. This could take the form of a primary research using both qualitative and quantitative methodologies to capture these factors and to assist in the validation of the transitional model.

## Supporting information

S1 File PRISMA Checklist. (DOCX)

S1 Fig Summary of themes. (ZIP)

## References

[1] World Health Organization. Primary health care, now more than ever. Geneva, Switzerland: 2008.

[2] World Health Organisation, editor Declaration of the Alm Ata Paper. International Conference on Primary Health Care; 1978 6–12 September; USSR.

[3] Betony K, Yarwood J. What exposure do student nurses have to primary health care and community nursing during the New Zealand undergraduate Bachelor of Nursing programme? Nurse Educ Today. 2013;33(10):1136–42. 10.1016/j.nedt.2012.12.007 23375697

[4] Henderson J, Javanparast S, MacKean T, Freeman T, Baum F, Ziersch A. Commissioning and equity in primary care in Australia: Views from Primary Health Networks. Health & social care in the community. 2018;26(1):80–9. 28608451 10.1111/hsc.12464

[5] Barrett A, Terry DR, Lê Q, Hoang H. Factors influencing community nursing roles and health service provision in rural areas: a review of literature. Contemporary nurse. 2016;52(1):119–35. 10.1080/10376178.2016.1198234 27264878

[6] Ashley C, Halcomb E, Brown A, Peters K. Experiences of registered nurses transitioning from employment in acute care to primary health care-quantitative findings from a mixed-methods study. Journal of Clinical Nursing. 2018;27(1–2):355–62. 10.1111/jocn.13930 28618208

[7] Jacelon C, Macdonald B, Fitzgerald F, Council Q. Reducing the rate of rehospitalization from postacute care: A quality improvement project. Rehabilitation Nursing. 2015;40(1):12–9. 10.1002/rnj.176 25308965

[8] Australian Health Ministers' Advisory Council. National Strategic Framework for Chronic Conditions. Canberra: Australian Government; 2017.

[9] Phelan A, McCarthy S, Adams E. Examining missed care in community nursing: A cross section survey design. Journal of advanced nursing. 2018;74(3):626–36. 10.1111/jan.13466 28960457

[10] Spoorenberg SL, Wynia K, Uittenbroek RJ, Kremer HP, Reijneveld SA. Effects of a population-based, person-centred and integrated care service on health, wellbeing and self-management of community-living older adults: A randomised controlled trial on Embrace. PloS one. 2018;13(1):e0190751 10.1371/journal.pone.0190751 29351295 PMC5774687

[11] World Health Organisation. Community health nursing report of a WHO expert committee (Technicla Report Series No. 558). Geneva: World Health Organisation, 1974. 4216177

[12] World Health Organization. Enhancing the role of community nursing for universal health coverage (Human Resources for Observer Series No. 18). Geneva, Switzerland: 2017.

[13] Freund T, Everett C, Griffiths P, Hudon C, Naccarella L, Laurant MJIjons. Skill mix, roles and remuneration in the primary care workforce: who are the healthcare professionals in the primary care teams across the world? 2015;52(3):727–43. 10.1016/j.ijnurstu.2014.11.014 25577306

[14] Homer CS, Passant L, Brodie PM, Kildea S, Leap N, Pincombe J, et al The role of the midwife in Australia: views of women and midwives. Midwifery. 2009;25(6):673–81. 10.1016/j.midw.2007.11.003 18276048

[15] Gray M, Malott A, Davis BM, Sandor C. A scoping review of how new midwifery practitioners transition to practice in Australia, New Zealand, Canada, United Kingdom and The Netherlands. Midwifery. 2016;42:74–9. 10.1016/j.midw.2016.09.018 27769012

[16] Tierney O, Sweet L, Houston D, Ebert L. A historical account of the governance of midwifery education in Australia and the evolution of the Continuity of Care Experience. Women and Birth. 2018;31(3):e210–e5. 10.1016/j.wombi.2017.09.009 29031648

[17] Kruske S, Kildea S, Jenkinson B, Pilcher J, Robin S, Rolfe M, et al Primary Maternity Units in rural and remote Australia: Results of a national survey. Midwifery. 2016;40:1–9. 10.1016/j.midw.2016.05.004 27428092

[18] Gao Y, Gold L, Josif C, Bar-Zeev S, Steenkamp M, Barclay L, et al A cost-consequences analysis of a Midwifery Group Practice for Aboriginal mothers and infants in the Top End of the Northern Territory, Australia. Midwifery. 2014;30(4):447–55. 10.1016/j.midw.2013.04.004 23786990

[19] Schwartz L, Wright D, Lavoie-Tremblay M. New nurses' experience of their role within interprofessional health care teams in mental health. Archives of Psychiatric Nursing. 2011;25(3):153–63. 10.1016/j.apnu.2010.08.001 21621729

[20] Speed S, Luker KA. Getting a visit: how district nurses and general practitioners ‘organise’each other in primary care. Sociology of health & illness. 2006;28(7):883–902. 17163858 10.1111/j.1467-9566.2006.00511.x

[21] King N, Ross A. Professional identities and interprofessional relations: evaluation of collaborative community schemes. Social Work in Health Care. 2003;38(2):51–72. 15022734 10.1300/j010v38n02_03

[22] Coddington R, Catling C, Homer CS. From hospital to home: Australian midwives’ experiences of transitioning into publicly-funded homebirth programs. Women and Birth. 2017;30(1):70–6. 10.1016/j.wombi.2016.08.001 27594344

[23] Commonwealth of Australia. National health reform agreement. In: National Health Funding Pool, editor. Canberra: Commonwealth of Australia; 2011.

[24] Jost SG, Bonnell M, Chacko SJ, Parkinson DL. Integrated primary nursing: A care delivery model for the 21st-century knowledge worker. Nursing administration quarterly. 2010;34(3):208–16. 10.1097/NAQ.0b013e3181e7032c 20562570

[25] Elnour AA, Dunbar J, Ford D, Dawda P. General practices’ perspectives on Medicare Locals’ performance are critical lessons for the success of Primary Health Networks. The Australasian medical journal. 2015;8(10):320 10.4066/AMJ.2015.2508 26576203 PMC4643609

[26] Powell Davies G, Harris M, Perkins D, Roland M, Williams A, Larsen K, et al Coordination of care within primary health care and with other sectors: a systematic review. 2017. 10.5694/j.1326-5377.2008.tb01748.x 18429740

[27] Sherman RO, Chiang‐Hanisko L, Koszalinski R. The ageing nursing workforce: a global challenge. Journal of Nursing Management. 2013;21(7):899–902. 10.1111/jonm.12188 24131080

[28] Halcomb E, Stephens M, Bryce J, Foley E, Ashley C. Nursing competency standards in primary health care: an integrative review. Journal of clinical nursing. 2016;25(9–10):1193–205. 10.1111/jocn.13224 26990487

[29] Halcomb E, Ashley C. Australian primary health care nurses most and least satisfying aspects of work. Journal of clinical nursing. 2017;26(3–4):535–45. 10.1111/jocn.13479 27461981

[30] Keleher H, Parker R, Francis K. Preparing nurses for primary health care futures: how well do Australian nursing courses perform? Australian Journal of Primary Health. 2010;16(3):211–6. 10.1071/PY09064 20815989

[31] McInnes S, Peters K, Hardy J, Halcomb E. Primary care clinical placements: the views of Australian registered nurse mentors and pre-registration nursing students (part 2). Nurse Education in Practice. 2015;15(6):443–9. 10.1016/j.nepr.2015.04.004 25960063

[32] Moher D, Liberati A, Tetzlaff J, Altman DG. Preferred reporting items for systematic reviews and meta-analyses: the PRISMA statement. Annals of internal medicine. 2009;151(4):264–9. 10.7326/0003-4819-151-4-200908180-00135 19622511

[33] Jackson N, Waters E. Criteria for the systematic review of health promotion and public health interventions. Health promotion international. 2005;20(4):367–74. 10.1093/heapro/dai022 16169885

[34] Popay J, Roberts H, Sowden A, Petticrew M, Arai L, Rodgers M, et al Guidance on the conduct of narrative synthesis in systematic reviews. A product from the ESRC methods programme Version. 2006;1:b92.

[35] PROSPERO. International prospective register of systematic reviews. 2016.

[36] Harvey C, Hegney D, Chamberlain D, Wirihana L, Sobolewska A, Wake T, et al Facilitating pathways to community nursing and midwifery: a systematic review. PROSPERO. 2018:CRD42018100228.

[37] Hegney D, Chamberlain D, Wirihana L, Harvey C, Sobolweska A, Knight B. From Incomer to Insider: A systematic review of the factors influencing the effective rapid and early career transition to a nursing speciality in different contexts of practice. BMC Nursing. Forthcoming. 10.1371/journal.pone.0216121 PMC6494050 31042747

[38] Barends E, Rousseau DM. Evidence-based management: How to use evidence to make better organizational decisions: Kogan Page Publishers; 2018.

[39] The Joanna Briggs Institute. Joanna Briggs Institute reviewers’ manual: 2014 edition. In: Joann, editor. The Joanna Briggs Institute Australia 2014.

[40] Austin L, Luker K, Martin R. Clinical nurse specialists and the practice of community nurses. Journal of advanced nursing. 2006;54(5):542–50. 10.1111/j.1365-2648.2006.03868_1.x 16722951

[41] Darvill A, Fallon D, Livesley J. A different world?: the transition experiences of newly qualified children’s nurses taking up first destination posts within children’s community nursing teams in England. Issues in comprehensive pediatric nursing. 2014;37(1):6–24. 10.3109/01460862.2013.855841 24490953

[42] Hunter B. Conflicting ideologies as a source of emotion work in midwifery. Midwifery. 2004;20(3):261–72. 10.1016/j.midw.2003.12.004 15337282

[43] McKenna H, Keeney S, Bradley M. The role of the community nurse in primary care led commissioning. Primary Health Care Research & Development. 2004;5(1):77–86.

[44] Patterson E, Price K, Hegney D. Primary health care and general practice nurses: what is the nexus? Australian Journal of Primary Health. 2005;11(1):47–54. 10.1071/PY05007

[45] Simons L, Lathlean J, Kendrick T. Community mental health nurses' views of their role in the treatment of people with common mental disorders. Primary Care Mental Health. 2006;4(2):121.

[46] Ashley C, Brown A, Halcomb E, Peters K. Registered nurses transitioning from acute care to primary healthcare employment: A qualitative insight into nurses' experiences. Journal of clinical nursing. 2018;27(3–4):661–8. 10.1111/jocn.13984 28771865

[47] Lazenbatt A, Orr J, Bradley M, McWhirter L. Community Nursing achievements in tackling inequalities in health in Northern Ireland. NT Research. 2000;5(3):178–92.

[48] Barlow A. Evaluation research: using comprehensive methods for improving healthcare practices. Evidence-Based Midwifery. 2004;2(1):4–8.

[49] Kemp L, Anderson T, Travaglia J, Harris E. Sustained nurse home visiting in early childhood: exploring Australian nursing competencies. Public Health Nursing. 2005;22(3):254–9. 10.1111/j.0737-1209.2005.220309.x 15982199

[50] Smith JC, Vandall-Walker V. A double whammy! New baccalaureate registered nurses' transitions into rural acute care. Rural & Remote Health. 2017;17:4256. 29284273 10.22605/RRH4256

[51] Aranda K. Community nurses’ talk of equality and the discursive constitution of selves. Journal of Advanced Nursing. 2005;51(2):131–9. 10.1111/j.1365-2648.2005.03476.x 15963184

[52] Fox R, Booker C., Turbutt A. Framework for lifelong learning for nurses and midwives. Brisbane: Queensland Health; 2018.

[53] Fox R, Burridge C, Bowen A, Curtis M, Firth S, Person N, et al Framework for lifelong learning In: Health Q, editor. Brisbane: metro North Hospital and Health Service; 2015.

[54] Wenger E. Communities of practice: Learning, meaning, and identity: Cambridge university press; 1999.

[55] Pesut DJ. The work of belonging. Journal of Nursing Scholarship. 2004;36(1):2–.

[56] Lea J, Cruickshank M. The role of rural nurse managers in supporting new graduate nurses in rural practice. Journal of Nursing Management. 2017;25(3):176–83. 10.1111/jonm.12453 27928887

[57] Price K, Patterson E, Hegney D. Being strategic: utilising consumer views to better promote an expanded role for nurses in Australian general practice. Collegian. 2006;13(4):16–21. 17285826 10.1016/s1322-7696(08)60535-1

[58] Procter N, Beutel J, Deuter K, Curren D, de Crespigny C, Simon M. The developing role of transition to practice programs for newly graduated mental health nurses. International Journal of Nursing Practice. 2011;17(3):254–61. 10.1111/j.1440-172X.2011.01932.x 21605265

[59] Gohery P, Meaney T. Nurses’ role transition from the clinical ward environment to the critical care environment. Intensive and Critical Care Nursing. 2013;29:321–8. 10.1016/j.iccn.2013.06.002 23886780

[60] Hartman SA. An Innovative Strategy for Community Nursing Student Simulation Experiences. Journal of Nursing Education. 2018;57(10):630–. 10.3928/01484834-20180921-13 30277552

[61] Bakon S, Craft J, Wirihana L, Christensen M, Barr J, Tsai L. An integrative review of graduate transition programmes: Developmental considerations for nursing management. Nurse education in practice. 2017 10.1016/j.nepr.2017.10.009 29045909

[62] Morphet J, Plummer V, Kent B, Considine J. A framework for transition to specialty practice programmes. Journal of Advanced Nursing. 2017;73(8):1970–81. 10.1111/jan.13279 28205248

[63] Fenwick J, Hammond A, Raymond J, Smith R, Gray J, Foureur M, et al Surviving, not thriving: a qualitative study of newly qualified midwives’ experience of their transition to practice. Journal of clinical nursing. 2012;21(13–14):2054–63. 10.1111/j.1365-2702.2012.04090.x 22672463

[64] Lea J, Cruickshank M. The experience of new graduate nurses in rural practice in New South Wales. Rural & Remote Health. 2007;7(4):814. 17958475

[65] Lea J, Cruickshank M. Supporting new graduate nurses making the transition to rural nursing practice: views from experienced rural nurses. Journal of Clinical Nursing. 2015;24(19–20):2826–34. 10.1111/jocn.12890 26177875

[66] Lea J, Cruickshank M. The support needs of new graduate nurses making the transition to rural nursing practice in Australia. Journal of clinical nursing. 2015;24(7–8):948–60. 10.1111/jocn.12720 25345730

