# Supplementary material for: Developing a community-based nursing and midwifery career pathway – A narrative systematic review
Source: PLoS One. 2019 Mar 28;14(3):e0211160. doi: 10.1371/journal.pone.0211160 (PMC6438448; doi:10.1371/journal.pone.0211160)
Supplement: S1 Fig — (ZIP) [file pone.0211160.s002.zip › Fig 4 Summary of Themes.docx]

Figure 4: Summary of themes

| **Themes** | **Sub-themes** | ***Enablers*** | **Inhibitors** |
| --- | --- | --- | --- |
| **PRE-ENTRY** |  |  |  |
| **Self** |  |  |  |
| ***Professional*** | Positive intention to community nursing | *I was excited about what opportunities were available. [46] (RN, refugee health)*  *I think just having the passion for PHC can make the transition better … it’s very rewarding. [46] (RN, refugee health)* |  |
|  | Confidence |  | *A new practitioner going into that* [PHC] *position. Unless they’re really sure of their scope of practice, to be able to push back and have the confidence. [46] (RN, GP)*  *As we* [students] *were coming up towards qualification, there was this big cloud stuck up here with the big word accountability written on it and that was quite scary. [41] (RN, NG, child health)* |
| **INCOMER** |  |  |  |
| **Self** |  |  |  |
| ***Professional*** | Competency | *Often there is nothing critical. If you put the wrong dressing on, it’s not going to really do any damage. [46] (RN, community health)*  *Sometimes if you into somebody’s house … there’ll be a whole line of relatives all wanting to watch and see what you’re doing so you’re used to performing in front of an audience. [41] (RN, NG, community health)* | *I felt like I had to ask … skilled colleagues … even just about the basic stuff … I felt like I should know more and I didn’t … I felt like I started afresh and I’m the new person again and it’s a bit annoying. [46] (RN, sexual health)*  *I was very concerned and worried for the immunisation component … I found* [it] *was very, very stressful having to work by yourself … having to learn in a short period of time when you’re under the pump and you’re very busy. Those are the times that things are going to happen. [46] (RN, GP)* |
|  | Feeling of isolation |  | *I was by myself in this role and I was like … so what am I supposed to do? I wasn’t sure of expectations. [46] (RN, school)*  *You’re on your own, you haven’t got any other nurses around you. [41] (RN, NG, child health)* |
|  | Recognition of past experience | *I didn’t feel out of my depth, I needed to just feel comfortable liaising with the [school] … it* [clinical skills] *just all naturally came back. [46] (RN, school)* | *The first home birth I went to she* [the mother] *had a baby in the water … and she was having a physiological* [third stage] … *I’d never seen one of those before. And the baby was born in the water and it took a good 30seconds to pink up and take its own breath. [22] (RM)*  *I put down what I have achieved in 3 years at University starting from scratch in community and not really being seen as being able to do anything … ‘you are the newly qualified’ … in a negative tone. [41] (RN, NG, child health)* |
|  | Role clarity |  | *Making sure I was informed enough to know what was my actual legislative requirements … because I felt so isolated on my own. [46] (RN, school)*  *I struggle with … the viewpoint of … what my health professional role is in general practice. [46] (RN, GP)*  *It was a surprise to me that somebody say to me, ‘you can’t make a diagnosis.’ …* [Another nurse] *went ‘oh that’s not our role’ … so I had a chat with a couple of GPs … It depends on the GP. [46] (RN, GP)*  *My scope of practice has changed considerably … in general practice everything is overshadowed by a doctor. [46] (RN, GP)* |
| ***Personal*** | Feeling confident | *I would have made errors …* [but] *where I’m working now is that you can’t really kill anybody. [46] (RN, sexual health)*  *It is a little bit scary when you go to your first one* [homebirth] *until you realise how normal it is. But … you’re still a midwife, you’ve got all the skills …* [in] *a different setting. [22] (RM)* | *I remember thinking oh boy, how am I ever going to remember any of this* [in community setting]*? [46]* *(RN, GP)*  *First* [time] *exposed to planned birth at home … we* [RM peer] *all go home and are thoroughly ill afterwards. [22] (RM)* |
| **Transition processes** |  |  |  |
| ***Formal*** | Length/duration of transition processes |  | *We still get called newly qualified nurses even though we’ve been qualified* [for] *seven months. [41] (RN, NG, child health)* |
|  | Orientation |  | *I was lucky to have two hours with the previous staff member* [who] *was going on maternity leave. [46] (RN, school)*  *I just turned up and found the key under the mat … because the other person had already left. It was very poorly managed. The booklet with the information wasn’t very clear or up to date. It was very vague and I had not much idea. [46] (RN, school)* |
|  | Orientation – Structure | *I put an orientation program together for the future … It’s very important in any workplace. If you orientate*[d] *well, you’ll have satisfied and content staff. [46] (RN, school)*  *That’s* [formal process of observation and assessment] *basically just trying to give us exposure to as many things as we can … it’s really good, really structured. [41] (RN, NG, child health)* | *They* [experienced RNs] *got nothing when they transitioned … thrown much more in the deep end. They were expected that* [as experienced RNs] *you should know what to do. [46] (RN, GP)* |
|  | Orientation – content | *A lot of our pathways … they are very quick … it is literally tick boxes which you can do. [41] (RN, NG, child health)* |  |
|  | Mentorship | *There was always someone there. I didn’t get left on my own until I was comfortable and confident in what I was doing. [46] (RN, GP)* |  |
|  | Supervision | *I was so nervous …* [when] *they put me down for visits on my own … one of our sisters … came with me and I was fine. [41] (RN, NG, child health)*  *I only went out when I felt ready and when everyone else was confident that I was able to do my job … and it just give you time to absorb everything and not having to go rush. [41] (RN, NG, child health)* | *I didn’t feel that I needed three months supervision … I am glad that I had somebody there but … I felt like I could have done somethings a bit sooner on my own. [41] (RN, NG, child health)*  *I was talking with my preceptor … about when to start IV training. She advised me not to do it until … after I had been qualified* [for] *a year. [41] (RN, NG, child health)*  *I’m a lot more relaxed doing stuff on my own, whereas if someone’s watching over me, I do become paranoid and I start stammering and do things wrong because I become very nervous. [41] (RN, NG, child health)* |
| ***Informal*** | Preceptorship | *We’ve got … one* [new midwife] … *who hasn’t been at homebirths before, then she’ll have an accredited midwife on who knows what’s going on. [22] (RM, manager)*  *Just because you’re deemed competent doesn’t meant that you can’t say … I am feeling a bit wobbly I need an extra person. That’s fine! [22] (RM, manager)* |  |
|  | Shadowing | *Because of induction process and because I was able to go out with more senior members of staff all the time it wasn’t that scary. [41] (RN, NG, child health)*  *I’m grad, because I have somebody there for support and do things right. [41] (RN, NG, child health)*  [as new graduate] *the first couple of weeks was mostly shadowing the practice nurses here … learning about diabetes health checks and what to look for, using the spirometry … things that I’ve never had to do before, nor learnt before. [46] (RN, GP)* | *It’s the time period where you’re going from being a student …where you were being supported, where you’re supernumerary … learning about the job … you’re still not quite independent and working on your own. [41] (RN, NG, child health)* |
|  | Self-directed learning |  | *There was just so much still to have to learn myself. [46] (RN, GP)* |
| **Sense of Belonging** | Support | *They* [peers] *were very good at making sure that I was okay … not feeling overwhelmed. [46] (RN, GP)*  *The staff are super-supportive. [46] (RN, sexual health)*  *It was a bit full on … wonderful people, wonderful doctors, wonderful manager, and wonderful support from that point of view. [46] (RN, GP)* | *You don’t always know, you can slip under the radar and just try to fumble your way. [46] (RN, remote area mental health)* |
| **CONTEXT SPECIFIC** |  |  |  |
| **New required skills** | Business model of practice | *It’s not so much they* [doctors] *don’t trust that we can do it, it’s just that to be able to get billed … a doctor has to be present. That’s very hard to comprehend … to be able to say you’re not really worth your opinion because a doctor’s opinion is the one that gets billed for. [46] (RN, GP)* | *You’ve got to pick up a patient load very quickly because there’s the need for the business. [46] (RN, GP)*  *Having to watch the money and having your day put into 15 minute blocks … getting used to the appointment system. [46] (RN, GP)*  *The biggest difference is the type of paperwork … the intensity of the record keeping was probably the most overwhelming thing at the beginning … it was how to work efficiently with the paperwork. [46] (RN, GP)* |
|  | Adapting to new practice setting | *In the hospital setting … the health care professionals have a lot of power and input over the patient’s care. Whereas when you are in someone’s home, obviously you want to empower the patients and the parents, you don’t let them intimidate you. [41] (RN, NG, child health)* | *A whole different ball game to working in the acute setting where you’ve got a controlled environment. [46] (RN, remote mental health)*  *I will never forget my first day … it was just a very different environment. [46] (RN, GP)* |
| **INSIDER** |  |  |  |
| **Self** |  |  |  |
| ***Professional*** | Competency | *You’re picking up signs that things could be physically wrong by what you’re asking them. [42] (RM)* |  |
|  | Autonomy | *The thing that sticks out most is the autonomy … you don’t have a doctor down the corridor. [41] (RN, NG, child health)* |  |
|  | Relationship with other professionals | *We do a lot of bouncing off each other, we all get together. Our doctors are very good. They rely on us a lot … the doctors will always* [say to patients] *‘these girls are the experts, go talk to them.’ [46] (RN, GP)* | *If she* [nonclinical practice manager] *had approached me in a different way I would have felt more respected, but right now I just feel disrespected. [46] (RN, GP)* |
|  | Job satisfaction | *Refugee Health is that area* [area I love]*, because theoretically and intellectually it stimulates me in so many different ways. [46] (RN, refugee health)*  *Being able to facilitate an entire experience from 20 weeks all the way through to 6 weeks postpartum with everything in the middle. I find that really satisfying actually. [22] (RM)*  *I had facilitated almost a thousand births … I had never seen a woman birth so calmly and physiologically as I did when I saw that woman birth at home. [22] (RM)*  *Families have just been so appreciative … and then you get to that point where they’ve cracked it and you can just see the relief in their faces and you feel like you’ve done a good job. [41] (RN, NG, child health)* |  |
|  | Ability to reflect own practice | *You have to be professional and be open minded at all times. [51] (District nurse practice educator)*  *One of the big things about birthing at home is I think you’re not on high alert because you’re less distracted with the goings of the hospital … you are more in-tune with what the woman’s body is doing. [22] (RM)*  *Part of my job as a midwife is protecting the birthing space and I feel that at home it’s a lot easier to do. [22] (RM)* |  |
|  | Support | *I enjoy having two* [midwives] *because you got a second pair of hands and a second perspective. [22] (RM)* | *It wasn’t to say that my mentor wasn’t around … when I was in the hospital surgical setting there’s only a set amount of things that a surgical nurse needs to do … in primary health I’m an immunisation nurse, a paediatric nurse, a geriatric nurse, a palliative care nurse, asthma, diabetes etc. [46] (RN, GP)* |
| ***Personal*** | Confidence | *By the end of the second week I was fine because I ended up doing quite a few shifts by myself … When I was on my own I had to make sure I knew what I was doing, and that turned out to be a really good thing. [46] (RN, school)*  *As a young nurse I learn fast, and the technology is my friend. Probably after about eight weeks I thought ‘okay, I can do this. I’m confident.’ [46] (RN, GP)* | *I think it takes a couple of years at least … to gain confidence as opposed to feeling comfortable in going out there. [46] (RN, remote area mental health)*  *When you feel that everything’s under control and you’re feeling okay about things then something happens … then suddenly you think ‘I wasn’t quite as sure as I thought I was.’ [46] (RN, GP)* |
| **BELONGING** |  |  |  |
| **Self** |  |  |  |
| ***Professional*** | Benchmarking | [Emotional rewording of working in community is] *the women! Getting to know them, them getting to know you! [42] (RM)*  *They* [women] *are the ones who keep you going. [42] (RM)*  *When you’re called at three o’clock in the morning … you don’t mind. [42] (RM)* | *It’s possible to spend a great deal of time doing things that wouldn’t be counted as work – the emotion bit can’t be measured. [42] (RM)* |
|  | Acknowledging acquired skills by other professionals | *I think that they* [GP] *are very happy to leave us to our own devices. [20] (district nurse, GP)* | *They* [GP] *are … unhappy when we ask them to look at thing that we* [nurses] *are not happy about, and that can cause conflict. [20] (district nurse, GP)*  *I referred someone for a visit recently, the doctor was really reluctant to go – he wanted me to give a prescription. I wanted the patient see, so I had to tell a white lie. [20] (district nurse, GP)*  *They* [GP] *make you look small in front of others … by intimating that you should have know*[n] *about it … sound like it is your fault rather than theirs for not telling you in the first place [20] (district nurse)* |
|  | Competency | *All of us* [community nurses] *within our team have got the leg ulcer course. So we feel quite competent and proficient to carry out the assessment. [40] (RN)*  *You’re going against your natural instinct wanting to help someone … when you try and encourage that person to help themselves but also lend a helping hand … the traditional role of a nurse is to be there to help. [45] (RN, mental health)* | *I think we* [community nurses] *are more comfortable with wounds … than continence … we do see a lot of incontinent patients, but they tend to be elderly people who* [we] *are just managing. But the young ones, that’s where I think we probably haven’t got the expertise. [40] (RN)*  *I’m not an expert in incontinence and they* [CNS] *are. They’ve got the expertise … I probably just think ‘pads’ … I’m too busy trying to contain that continence. [40] (RN)*  [You] *wouldn’t have time wasted with seeing people who just don’t fit the criteria* [for community mental health service]*. [45] (RN, mental health)* |
|  | Knowledge/background needed | *I see psychosis as something that will come through* [to the community mental health team] *and it will be common to our service. [45] (RN, mental health)*  *Basically patients who should be in our service are these guys with severe mental illnesses for a long period of time. [45] (RN, mental health)*  *The nature of mental health, it is very difficult to draw boundaries. [45] (RN, mental health)*  *The stress associated with social disadvantage and in particular the misery of postnatal depression were an everyday reality when I worked as a health visitor. [47] (RN, child protection specialist)*  *The significance of childbirth and the mental health of women who live in deprived communities requires the proactive stance taken by nursing in collaboration with a range of other professionals to tackle these inequalities and promote the application of evidence-based information in clinical practice through audit and evaluation. This is an area where we can contribute significantly by providing responsive, client-centred care underpinned by evidence of effectiveness and based on a preventive model. [47] (nurse specialist)* | *It can be really frustrating working under … limiting* [strict] *guidelines [22] (RM)* |
|  | Commissioning roles | *Some community nurses will be able to take a lead role in commissioning. It is more about the right person in the right place at the right time. [43] (Director of Nursing, Health and Social Services Trust)*  *The person who takes the lead* [commissioning] *role should be the person who … has the most skill and the most knowledge. [43] (Director of Nursing, Health and Social Services Trust)*  *Community nurses can take a very major role in commissioning. It would be a mistake to think that nurses were the only people involved in commissioning. [43] (Chief Nurse, Health and Social Services Boards)*  *Nurses are as well qualified as anyone else to take on this role. [43] (Senior Nurse Manager, Health and Social Services Executive)* | *People* [nurses] *who have been community based for many years will need some exposure to the acute sector* [to take lead role in commissioning]. *[43] (Director of Nursing, Health and Social Services Trust)*  *The difficulty with community nurses is that their education and their exposure may have been limited for the broader commissioning requirements. [43] (Chief Executive, Health and Social Services Board)*  *One of the issues is the knowledge and skills required to commission and lead services … Community nurses have a role to play … but development work on skills and knowledge must take place. [43] (senior nurse educator)*  *I think there are people who have* [skills and knowledge about commissioning]*, need significant investment in developing these skills. [43] (Chief Executive, health and Social Service Board).*  *Need to be honed up* [these skills]*, particularly negotiation skills. [43] (Director of Nursing)* |
|  | Future aspiration | [Recent change at local and national levels] *is exciting and stimulating; that’s what I want to be involved with rather than washing people and getting them up. [21] (district nurse)* |  |
| **Transition processes** |  |  |  |
| **Formal** | Continuous Professional Development | *Brilliant … I’ve been very fortunate* [accessing continuous development]*. It’s always supportive. [46] (RN, refugee health)*  *We’ve been lucky about that sort of thing. The funding has been available and they’ve been very supportive. [46] (RN, remote area mental health)*  *We do two* [homebirth drills] *a year. [22] (RM)* | *Trying to get education is extremely difficult. We have to ask every single time and plead our case and virtually prove it would be beneficial. Just getting my CPR done … it’s going to be up to me financially … to pay for all these things. [46] (RN, school)*  *I don’t even ask them to pay for me to study—I just asked them to have study leave and my manager emailed me back saying, ‘sorry we’re not interested.’ [46] (RN, GP)*  *I was letting the practice down for not being on the floor, and then they wouldn’t cover it. [46] (RN, GP)*  *I think we would be at risk of losing very skilled and valuable members of the community mental health team and I think that happens when new initiatives come up (development of primary healthcare role). [45] (RN, mental health)* |
| **Sense of Belonging** |  |  |  |
|  | Relationship with other professionals | *I don’t think they* [community nurses] *have been deskilled because … they can now do* [more] *because of me* [CNS] *being in post and feeding information and training to enable them to do a full leg ulcer assessment. [40] (CNS, tissue viability nurses)*  *We* [CNS] *are facilitators of care not providers of care … it is a partnership with them* [community nurses]*. [40] (CNS, continence advisor)*  *They* [CNS]*’re there as a resource and communication and education … without specialist input we* [community nurses] *wouldn’t be able to lift our heads up. [40] (RN)*  *With the assistance of a multidisciplinary team comprising obstetricians, midwives, physiotherapists, and a social worker, we can now prepare mothers for parenthood. [47] (RM)*  *We save them* [GP] *a lot of work, we do things for them that are really not our job, taking messages for them, checking up on things, doing bloods. [20] (district nurse, GP)*  [We practice nurses] *accommodate their* [GPs] *individual needs and wants. [44] (Practice nurse, GP)*  *When you* [district nurse] *are attached to a GP and you are their nurse then … you generally save them a lot of work, we manage people for them and save them having to do a lot of unnecessary visits really with them, we decide for them and then they rubber stamp things. [20] (district nurse, GP)*  [In a situation where patient was refusing treatment] *The nurse met the doctor and asked to see her* [the patient]. *She* [the nurse] *outlined the problem and the doctor listened carefully and intently … They discussed their different methods and at the end of discussion, the doctor said ‘let’s both go away and think about it and then we will meet at 1pm.’ … I was struck by how atypical this encounter was. [20] (RN, GP)*  *Usually you have got everything set up and ready and you’ve probably already done a few things depending on what has happened so it save them a lot of time and energy. [44] (Practice nurse, GP)*  *You often don’t realise how or why you do things the way you do until someone picks you up on it … that constructive criticism, in a really friendly, loving way, meant to help you improve. [22] (RM)* | *This is supposed to be my case load. I’m supposed to be responsible for these women, and yet I’ve got somebody* [other professionals] *up there saying ‘oh, no, you can’t do that’. And that’s very frustrating, and I feel that they’re not respecting my position – because I’m not giving these women what they want … very frustrating and I think will be the ruin of midwifery, really. [42] (RM)*  *They* [community nurses] *just couldn’t wait to get rid of them* [incontinence] *… I could see that by the information we never got back, they hated doing continence assessments, and the only thing they could ever do with the assessment is give pads as a way out, it was never looked at constructively of ‘why are you incontinent?’ [40]* *(CNS, continence advisor)*  *The challenging aspect is dealing with the practice manager that doesn’t have a clinical background. [46] (RN, GP)*  *It’s hard for her* [practice manager] *to do a performance review. She doesn’t know what you do, and the GPs wouldn’t have the time … it’s not their area. [46] (RN, GP)*  *A negative comment* [from the doctor] *that I was employed as an RN and I’m to do what I’m told to do. [46] (RN, GP)* |
|  | Workplace violence/Power relationship |  | *Occasionally when you have been to speak to a doctor and they have made you feel stupid … make you feel a bit shitty. [20] (district nurse, GP)*  *I don’t stand for it* [accept unreasonable behaviour from doctors] *really, I am always getting into trouble with him* [GP] *and the Trust. [20] (district nurse, GP)*  *You have to meet them* [daily demands, rules and routines of communication] *and respond to their* [GPs] *referrals as they make them and make sure you are sure about visits … if you don’t they complain. [20] (RN, GP)* |
|  | Lifelong care – beyond individual patient | [My most important function is] *getting to know people, families, really well; making a real difference to their overall wellbeing. [44] (Practice Nurse, Health centre)*  *I think you do more health promotion and counselling here* [in community] *than … in a hospital. I have to think more about their* [patient’s] *whole life and how things affect their families. [44] (Practice Nurse)*  *I’ve looked after some kids from when I immunised them to when they’ve come about their acne. [44] (Practice Nurse)*  *I’ve seen some women through childbirth to menopause. [44] (Practice Nurse)*  *You know you’re making a bit of difference … even a child sleeping through the night with eczema, it’s making a difference to parents. [41] (RN, NG, child health)* |  |
|  | Remuneration | *Equal pay of an equal job. Unquestioningly. [43] (Director of Nursing)* | [If community nurses are commissioning] *in principle this* [equally remunerated to GPs] *should happen …* [However] *nurses are employees whereas GPs are independent contractors. [43] (Senior nurse manager)*  *They* [GPs] *would be paid significantly for what they do but I would not see an equal remuneration. [43] (Senior nurse manager)* |
| ***Support*** | Lack of managerial support |  | *We get lots of support from each other but not from our managers. They will not support you at all, they are too worried about* [their own] *contracts. [20] (RN, GP)*  *I just carry on as best as I can … nurse my patients and try to fit in with what is going on around me, but … not being able to do the work is a treat. [20] (RN, GP)* |
| **CONTEXT SPECIFIC** |  |  |  |
| **Organisational support – policies/guidelines** | Role and boundary | *They* [RM in hospital and home setting] *don’t do anything differently; they follow the same guidelines. [22] (RM, manager)*  *I do a reasonable amount of health promotion on the campus. I try and highlight two to three issues a semester … I also write articles* for the student newspaper]*. [44] (Practice Nurse, Health centre)*  [I do everything from] *a top to toe health assessment to making sure kids have a proper lunch for school. [44] (Practice nurse, health centre)*  *There was this whole band of people who were really very distressed and really quite unwell at times who wouldn’t have got any support, apart from popping along to their GP … who equally were not appropriate for community mental health team. [45] (RN, mental health)*  *The argument that when people first present with a depression or an anxiety, that they actually do get some kind of short focus treatment … it stops them coming into our system. [45] (RN, mental health)* | *I feel* [social service is] *totally out of my role. They* [stakeholders] *have skimmed off the top, I feel left with the odd jobs. [21] (district nurse)*  *They* [i.e. patients cared for on the scheme] *shouldn’t be taking beds up in the hospital I agree to that. They should be put into … social services, we* [district nurses] *shouldn’t have to sit with them … and they* [social services] *are not footing the bill! [21] (district nurse)*  *I think social services are abusing it* [i.e. Fast Response scheme] *– it’s an easy way of getting cover and they are not footing the bill. [21] (district nurse)* |
|  | Funding |  | [Public-funded home birth model] *is actually quite a difficult model to work in. There’s still a great deal of resistance between hospital and home birth … If you need to transfer a woman, you never know what you were going to get. [22] (RM)* |
